# 5-Hydroxytryptamine-3 receptor antagonist and dexamethasone as prophylaxis for chemotherapy-induced nausea and vomiting during moderately emetic chemotherapy for solid tumors: a multicenter, prospective, observational study

**DOI:** 10.1186/s40360-020-00445-y

**Published:** 2020-10-06

**Authors:** Reiko Matsui, Kenichi Suzuki, Tomomi Takiguchi, Makoto Nishio, Takeshi Koike, Toshinobu Hayashi, Takashi Seto, Yuki Kogure, Naoyuki Nogami, Kimiko Fujiwara, Hiroyasu Kaneda, Tomohiko Harada, Satoru Shimizu, Masashi Kimura, Hirotsugu Kenmotsu, Mototsugu Shimokawa, Koichi Goto

**Affiliations:** 1 Department of Pharmacy, National Cancer Center Hospital East, 6-5-1, Kashiwanoha, Kashiwa-shi, Chiba, 277-8577 Japan; 2 Department of Pharmacy, Japanese Foundation for Cancer Research, Cancer Institute Hospital, Tokyo, Japan; 3 Hoshi University Division of Applied Pharmaceutical Education and Research, 2-4-41 Ebara, Shinagawa-Ku, Tokyo, 142-8501 Japan; 4 Department of Thoracic Medical Oncology, Japanese Foundation for Cancer Research, Cancer Institute Hospital, 3-8-31, Ariake, Koto, Tokyo, 135-8550 Japan; 5 Department of Pharmacy, Clinical Research Institute, National Hospital Organization, Kyushu Cancer Center, 3-1-1 Notame, Minami-ku, Fukuoka, 811-1395 Japan; 6 Department of Pharmaceutical and Health Care Management, Faculty of Pharmaceutical Sciences, Fukuoka University, 8-19-1 Nanakuma, Jonan-ku, Fukuoka, 814-0180 Japan; 7 Department of Thoracic Oncology, Clinical Research Institute, National Hospital Organization, Kyushu Cancer Center, 3-1-1 Notame, Minami-ku, Fukuoka, 811-1395 Japan; 8 Department of Pharmacy, National Hospital Organization, Shikoku Cancer Center, 160, Minamiumemoto-machi-kou, Matsuyama-city, Ehime 791-0280 Japan; 9 Department of Thoracic Oncology, National Hospital Organization, Shikoku Cancer Center, 160, Minamiumemoto-machi-kou, Matsuyama-city, Ehime 791-0280 Japan; 10 Department of Pharmacy, Kindai University Hospital, 377-2 Ohno-higashi, Osaka-Sayama, Osaka, 589-8511 Japan; 11 Department of Medical Oncology, Kindai University Hospital, 377-2 Ohno-higashi, Osaka-Sayama, Osaka, 589-8511 Japan; 12 Department of Clinical Oncology, Osaka City University Graduate School of Medicine, 1-4-3, Asahimachi, Abeno-ku, Osaka, 545-8585 Japan; 13 Department of Pharmacy, Kanagawa Cancer Center, 2-3-2, Nakao, Asahi-ku, Yokohama-shi, Kanagawa 241-0815 Japan; 14 Department of Breast and Endocrine Surgery, Kanagawa Cancer Center, 2-3-2, Nakao, Asahi-ku, Yokohama-shi, Kanagawa 241-0815 Japan; 15 Department of Pharmacy, Shizuoka Cancer Center, 1007 Shimonagakubo, Nagaizumi-cho, Sunto-gun, Shizuoka, 411-8777 Japan; 16 Division of Thoracic Oncology, Shizuoka Cancer Center, 1007 Shimonagakubo, Nagaizumi-cho, Sunto-gun, Shizuoka, 411-8777 Japan; 17 Department of Cancer Biostatistics Laboratory, Clinical Research Institute, National Hospital Organization, Kyushu Cancer Center, 3-1-1 Notame, Minami-ku, Fukuoka, 811-1395 Japan; 18 Department of Thoracic Oncology, National Cancer Center Hospital East, 6-5-1, Kashiwanoha, Kashiwa-shi, Chiba, 277-8577 Japan

**Keywords:** Antiemetic therapy, Chemotherapy-induced nausea and vomiting (CINV), Moderate emetic risk chemotherapy (MEC), NK-1 receptor antagonist, 5-HT3 receptor antagonist

## Abstract

**Background:**

Of patients receiving moderate emetic risk chemotherapy (MEC), 30–90% experience chemotherapy-induced nausea and vomiting (CINV); however, the optimal antiemetic treatment remains controversial.

**Methods:**

In this multicenter, prospective, observational study of adults treated with MEC while receiving chemotherapy for various cancer types in Japan, the enrolled patients kept diaries documenting CINV. All participants received a 5-hydroxytryptamine-3 receptor antagonist and dexamethasone.

**Results:**

Of the 400 patients enrolled from May 2013 to January 2015, 386 were eligible for evaluation. The median age was 64 (range, 26–84). The overall complete response (CR; no emetic events and no antiemetic measures) rate was 64%. The proportion of patients showing CR was low in the carboplatin (CBDCA)- and oxaliplatin-based chemotherapy groups, especially among women. We showed that the CR rates in men were high in the CBDCA (AUC5) + etoposide (ETP) (80%), capecitabine plus oxaliplatin (CAPOX) (78%), and CBDCA+ paclitaxel (PTX) groups for lung cancer (73%). Total control (TC; no emetic events, no antiemetic measures, and no nausea) and complete control (CC; no emetic events, no antiemetic measures, and less than mild nausea) were achieved in 51 and 61% of patients, respectively. Logistic regression analysis revealed history of motion sickness, history of pregnancy-associated vomiting and CBDCA-based chemotherapy as risk factors for CR and history of motion sickness and history of pregnancy-associated vomiting as risk factors for TC. Additional, Ages ≥65 years is an independent predictive factor for achieving TC.

**Conclusions:**

Our data showed that two antiemetics were insufficient to control CINV in patients receiving CBDCA- or oxaliplatin-based chemotherapy. However, two antiemetics may be sufficiently effective for elderly male patients receiving CBDCA (AUC5) + ETP, CBDCA+PTX for lung cancer, or CAPOX. Additionally, we consider that three antiemetics are necessary for women with colorectal cancer receiving CAPOX. Risk factor analysis related to CR showed that CINV prophylaxis in patients treated with CBDCA-based chemotherapy was generally supportive of the guideline-recommended three antiemetics. However, the control of nausea in patients receiving non-CBDCA-based chemotherapy is a key point to note. The further individualization of antiemetic regimens for patients receiving MEC based on both types of chemotherapy regimens and sex is needed.

## Background

Chemotherapy-induced nausea and vomiting (CINV) are major adverse effects of cancer chemotherapy that impair the quality of life of patients and often cause a delay in or refusal of potentially curative chemotherapy. Over the past decade, antiemetic treatment has been greatly improved by the development of new antiemetic agents and international guidelines for antiemetic therapy prepared by well-known organizations, such as the American Society of Clinical Oncology (ASCO) [1], the Multinational Association of Supportive Care in Cancer (MASCC)/European Society of Medical Oncology (ESMO) [2], and the National Comprehensive Cancer Network (NCCN) [3]. The Japanese guidelines for CINV published in 2010 recommend two antiemetics for moderate emetic risk chemotherapy (MEC): 5-hydroxytryptamine-3 receptor antagonist (5HT3RA) and dexamethasone. However, both the Japanese and international guidelines recommend a three-drug combination of a neurokinin 1 receptor antagonist (NK1RA), a 5HT3RA, and dexamethasone for patients receiving carboplatin, which is classified as MEC. However, whether the addition of an NK1RA to a 5HT3RA and steroid combination is beneficial in patients receiving MECs other than carboplatin-based regimens remains controversial. Providing a single recommendation for antiemetic treatment for the entire broad range of expected CINV in the moderate level (30–90%) is problematic. Furthermore, the recommendations of international guidelines are based on the emetic potential of anticancer agents when given in the absence of antiemetic prophylaxis and show minimal consideration for patient-related factors. Few studies have compared the incidence of CINV for different MEC regimens. Different antiemetic treatment strategies may be optimal for different regimens and risk factors. Risk factors reportedly associated with CINV include younger age, female sex, history of CINV, and low alcohol consumption for several solid tumors [4–6]. The identification of risk factors for CINV is important for the selection of the appropriate care for various MEC regimens. Despite advances in prophylactic antiemetics, many aspects of CINV in MEC remain unclear. Despite the existence of various regimens in MEC, the antiemetic guidelines are only divided into CBDCA-based chemotherapy and non-CBDCA-based chemotherapy. The purpose of this study was to investigate the need for individualization of antiemetic treatments for different chemotherapeutic regimens and MEC risk factors. The study focused on clarifying poor control regimens of CINV and risk factors associated with CINV for MEC.

## Methods

### Study design

This was a multicenter, prospective, observational study in which seven institutions throughout Japan participated. We selected National Cancer Center Hospital East, Cancer Institute Hospital, Kyushu Cancer Center, Shikoku Cancer Center, Kindai University Hospital, Kanagawa Cancer Center, and Shizuoka Cancer Center and asked them to participate in this study. All procedures were performed in accordance with the ethical standards of the institutional and/or national research committee and the 1964 Helsinki Declaration and its later amendments or comparable ethical standards. This study was approved by the National Cancer Center Institutional Review Board (2012–324) and the Institutional Review Board of each participating hospital.

### Enrollment of patients

Written informed consent was obtained from all participants prior to registration. The primary inclusion criteria were ≥ 20 years of age, diagnosis of solid tumors, no prior chemotherapy, and planned administration of combination therapy with a 5-HT3 receptor antagonist and dexamethasone. The combinations administered were as follows: for lung cancer, carboplatin plus etoposide (CBDCA+ETP), carboplatin plus paclitaxel (CBDCA+PTX), or carboplatin plus pemetrexed therapy (CBDCA+PEM); for breast cancer, cyclophosphamide plus docetaxel therapy (DTX + CPA); for colon cancer: oxaliplatin with fluorouracil and folinic acid chemotherapy (FOLFOX) or capecitabine plus oxaliplatin (CAPOX); and for ovarian cancer, carboplatin plus paclitaxel therapy (CBDCA+ PTX). The exclusion criteria were patients with gastrointestinal obstruction, ascites or pleural effusion, or symptomatic brain metastasis, and those receiving current radiotherapy directed toward the abdomen/pelvis.

Based on the feasibility of each facility, we aimed to register 50 patients per regimen, with 150 colorectal cancer cases, 150 lung cancer cases, 50 ovarian cancer cases, and 40 breast cancer cases per regimen.

### Management of patient diaries and collection of required data

Before initiating cancer chemotherapy, patients were provided with 7-day diaries to record their CINV symptoms. They were asked to record digestive symptoms, such as the development and severity of nausea, frequency of vomiting, and number of salvage treatments, including the use of antiemetic medications (e.g., metoclopramide, domperidone, and olanzapine). Nausea was assessed by patients themselves using the 4-point Likert Scale (0: No Nausea, 1: Mild, 2: Moderate, and 3: Severe), and the results were recorded in their daily diaries. Patients were required to write their symptoms in the diary every day for 7 days from the initiation of their anticancer MEC. The investigators and/or their colleagues recorded background patient information, including sex, age, treatment history (history of radiotherapy, use of anticancer drugs, or use of anxiolytic drugs before administration of the anticancer drug), alcohol intake history, smoking history, risk factors for CINV (history of motion sickness or vomiting related to pregnancy), performance status, cancer chemotherapy regimen (type and dose of drug and timing of administration), and details of antiemetic therapy and salvage treatment for CINV extracted from the patients’ diaries. The patients were requested to fill in their diaries and hand them over to the person in charge of this study at the end of the observation period. The diaries were also sent to the secretariat by the investigators after the extraction of the required data.

### Antiemetic regimen

Patients received a guideline-based combination of a 5HT3RA and dexamethasone for MEC. The dosage of 5HT3RA was either palonosetron in 0.75 mg and a first-generation 5HT3RA in standard doses. The dose of dexamethasone was in accordance with each institution’s policy. On day 1 of chemotherapy (acute phase), 5HT3RA and dexamethasone were administered. Day2–3of after chemotherapy (delayed phase), dexamethasone was in accordance with each institution’s policy.

### Outcomes

The objective of this study was to evaluate the incidence of CINV for different MEC regimens based on the complete response (CR), total control (TC), and complete control (CC) rates over the entire observation period (0–168 h), acute phase (0–24 h), and delayed phase (24–168 h) of the first cycle of treatment. We also aimed to evaluate the emetic event rate over the entire observation period and time to treatment failure.

The CR rate was defined as the proportion of participants in the analysis set with no emetic events and no antiemetic measures, the TC rate as the proportion of participants with no emetic episodes, no antiemetic measures, and no nausea, and the CC rate as the proportion of participants with no emetic episodes, no antiemetic measures, and less than mild nausea. The severity of nausea was measured using a 4-point Likert Scale. Time to treatment failure was defined as the time to the first emetic episode or use of rescue medications.

Risk factors associated with good control of CINV were assessed for sex, age, motion sickness, drinking habit, smoking history, pregnancy, type of 5HT3RA and CBDCA-chemotherapy, or others associated with CR and TC.

### Data analysis

Patient characteristics and CR, TC, and CC rates were summarized using descriptive statistics or contingency tables. Independent risk factors for CR and TC were analyzed using univariate logistic regression. The number of risk factors for CINV in the collected data sets was analyzed by multivariate logistic regression analysis with the backward elimination method. The following independent factors were included in the model: sex, age, motion sickness, drinking habit, smoking history, pregnancy-associated vomiting, and the type of 5HT3RA and CBDCA-based chemotherapy. For all analyses, *p*-values correspond to two-sided tests, and *p* < 0.05 was considered to indicate statistical significance. All statistical analyses were performed using SAS 9.2.

## Results

### Patient selection and characteristics

**Fig. 1:**
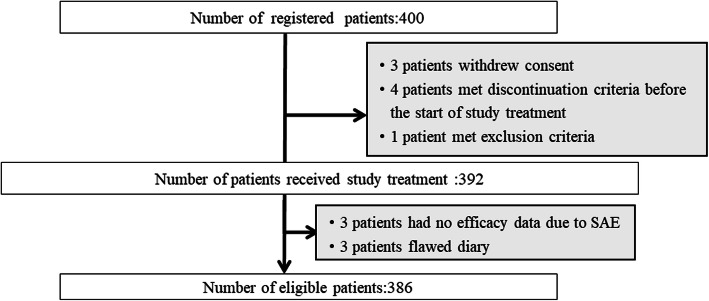
Flow chart showing the enrollment of patients

Between May 2013 and January 2015, 400 patients were registered in this study, 386 of whom were eligible for evaluation. Fourteen patients were excluded from analyses for the following reasons: three withdrew consent, four met the discontinuation criteria before the start of the study treatment, one met the exclusion criteria, three lacked efficacy data because of serious adverse effects, and three had not completed their diary correctly (Fig. 1). Table 1 summarizes the characteristics of eligible patients. Patients with breast cancer and ovarian cancer were almost exclusively female and younger than those with other malignancies. Patients with other malignancies were on average older and predominantly male. Only four patients received the FOLFIRI regimen because this is mainly used as second-line chemotherapy, thus not fulfilling the enrolment criteria. Table 2 summarizes the antiemetic regimen. The median dose of dexamethasone was 9.9 mg (6.6–19.8 mg) in the acute phase and 4 mg (2–10 mg) in the delayed phase. 5-HT3RA was administered at standard doses.

**Table 1:** Patient characteristics

	All Patients (*n* = 386)	Colorectal cancer (*n* = 161)	Lung cancer (*n* = 140)	Ovarian cancer (*n* = 45)	Breast cancer (*n* = 40)
Age,n (%) ≧65 years	178 (46.1)	66 (41.0)	92 (65.7)	9 (20.0)	11 (27.5)
< 65 years	208 (53.9)	95 (59.0)	48 (34.3)	36 (80.0)	29 (72.5)
Median (range)	64 (26–84)	62 (30–79)	68 (39–84)	57 (26–76)	54.5 (37–73)
Sex (%) Female	185 (47.9)	71 (44.1)	29 (20.7)	45 (100.0)	40 (100.0)
Motion sickness (%) Yes	59 (15.3)	22 (13.7)	12 (8.6)	13 (28.9)	12 (30.0)
Drinking habit (%) Yes	203 (52.6)	79 (49.1)	83 (59.3)	23 (51.1)	18 (45.0)
Smoking habit (%) Yes	150 (38.9)	56 (34.8)	88 (62.9)	2 (4.4)	4 (10.0)
Pregnancy associated vomiting (%) Yes	95 (51.4)	34 (47.9)	20 (69.0)	20 (44.4)	21 (52.5)
Palonosetoron (%)	153 (39.6)	132 (82.0)	9 (6.4)	1 (2.2)	11 (27.5)
Regimen					
CBDCA (AUC5) + ETP	34 (8.8)	–	34 (24.3)	–	–
CBDCA+PTX	103 (26.7)	–	58 (41.4)	45 (100.0)	–
CBDCA (AUC5)	20	–	2	18	–
CBDCA (AUC6)	83	–	56	27	–
CBDCA+PEM	48 (12.4)	–	48 (34.3)	–	
CBDCA (AUC5)	11	–	11	–	–
CBDCA (AUC6)	37	–	37	–	–
DTX + CPA	40 (10.4)	–	–	–	40 (100.0)
FOLFOX	79 (20.5)	79 (49.1)	–	–	–
FOLFIRI	4 (1.0)	4 (2.5)	–	–	–
CAPOX	78 (20.2)	78 (48.4)	–	–	–

*CBDCA* Carboplatin, *ETP* Etoposide, *PTX* Paclitaxel, *PEM* Pemetrexed, *CPA* Cyclophosphamide, *DTX* Docetaxel, *FOLFOX* oxaliplatin with fluorouracil and folinic acid, *FOLFIRI* Irinotecan with fluorouracil and folinic acid, *CAPOX* Capecitabine plus oxaliplatin therapy

**Table 2:** Antiemetic regimen

	5HT3RA			N(%)	Dexamethasone	N(%)
**FOLFOX**	Palonosetron	Day1		60 (75.9)	Day1	4 (6.7)
					Day1–3	56 (93.3)
	1st generation 5HT3RA	Day1		19 (24.1)	Day1	7 (36.8)
					Day1–3	12 (63.2)
**CAPOX**	palonosetron	Day1		72 (92.3)	Day1	2 (2.8)
					Day1–3	70 (97.2)
	1st generation 5HT3RA	Day1		6 (7.7)	Day1	2 (33.3)
					Day1–3	4 (66.7)
**CBDCA + ETP**	1st generation 5HT3RA	Day1		34 (100.0)	Day1–3	34 (100.0)
**CBDCA + PTX**	1st generation 5HT3RA	Day1		58 (100.0)	Day1	57 (98.3)
**(Lung Cancer)**					Day1–3	1 (1.7)
**CBDCA + PEM**	palonosetron	Day1		9 (18.7)	Day1	7 (77.8)
					Day1–3	1 (11.1)
					Day1–4	1 (11.1)
	1st generation 5HT3RA	Day1		39 (81.3)	Day1	31 (79.5)
					Day1–2	1 (2.6)
					Day1–3	7 (17.9)
**CBDCA + PTX**	palonosetron	Day1		1 (2.2)	Day1	1 (2.2)
**(Ovarian Cancer)**	1st generation 5HT3RA	Day1	42	44 (97.8)	Day1	33 (73.3)
		Day1–3	2		Day1–3	11 (24.4)
**DTX + CPA**	palonosetron	Day1		11 (27.5)	Day1	5 (45.5)
					Day1–3	1 (9.1)
					Day1–4	5 (45.5)
	1st generation 5HT3RA	Day1		29 (72.5)	Day1	1 (3.4)
					Day1–3	1 (3.4)
					Day1–4	27 (93.1)

*5HT3RA* 5-hydroxytryptamine-3 receptor antagonist, *FOLFOX* Oxaliplatin with fluorouracil and folinic acid

*CAPOX* Capecitabine plus oxaliplatin therapy, *CBDCA* Carboplatin, *ETP* Etoposide, *PTX* paclitaxel

*PEM* Pemetrexed, *DTX* Docetaxel, *CPA* Cyclophosphamide

### Control of CINV

**Fig. 2:**
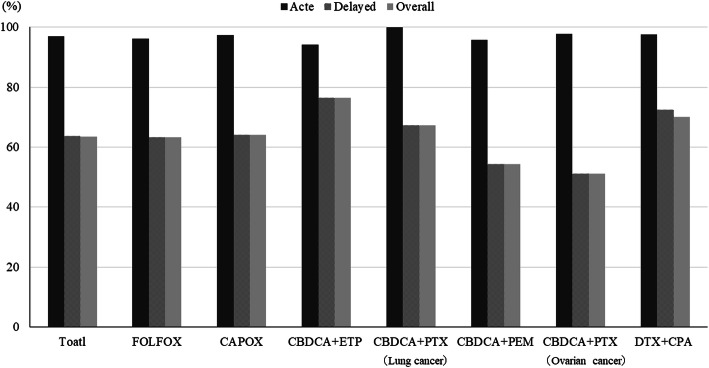
Complete response rates

**Fig. 3:**
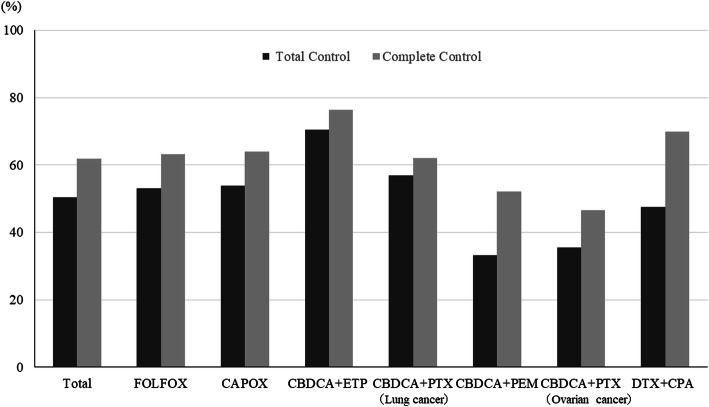
Total control rate and complete control rate during the overall phase

**Fig. 4:**
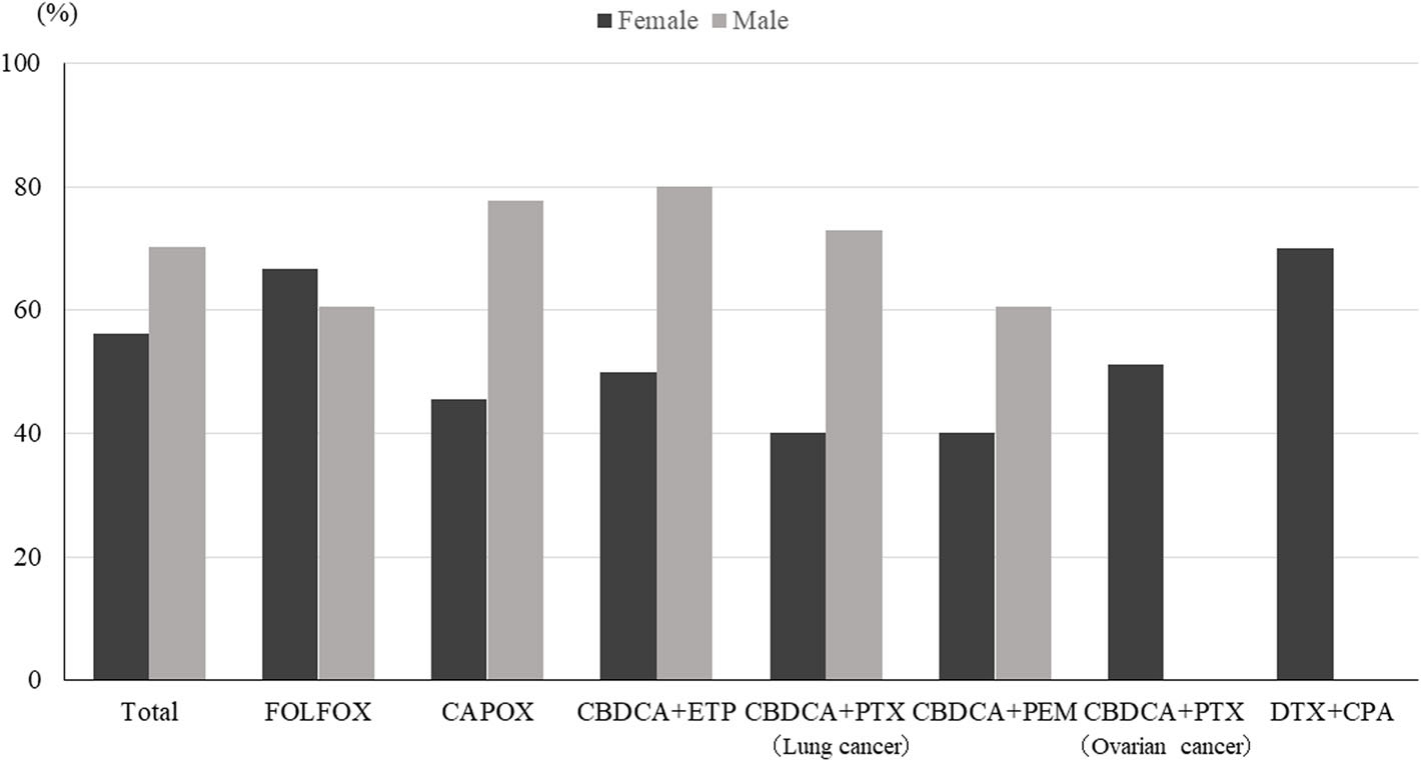
Complete response rate during the overall phase

CR rates was achieved by 64% of patients over the entire observation period (0–168 h), the details being as follows: FOLFOX, 63% and CAPOX, 64% for colon cancer; CBDCA+ETP, 77%, CBDCA+PTX, 67%, and CBDCA+PEM, 54% for lung cancer; CBDCA+PTX, 51% for ovarian cancer; and DTX + CPA, 70% for breast cancer (Fig. 2). The proportions were similar for the overall and delayed phases. Of the patients who received MEC regimens, 51 and 61% achieved TC and CC, respectively, over the entire observation period (Fig. 3). The overall CR rate for CAPOX occurred significantly more frequently in men (78%) than in women (46%). We showed that the overall CR rates in men were high in CBDCA+ETP (80%), CAPOX (78%), and CBDCA+PTX groups for lung cancer (73%)(Fig. 4). The overall CR rate for CBDCA-base chemotherapy was 61.6%, and non-CBDCA-base chemotherapy was 65%. The overall CR rate for CBDCA-base chemotherapy occurred significantly more frequently in men (71.2%) than in women (47.3%), but non-CBDCA-based chemotherapy was no significant difference between men (68.9%) and women (62.2%).

**Fig. 5:**
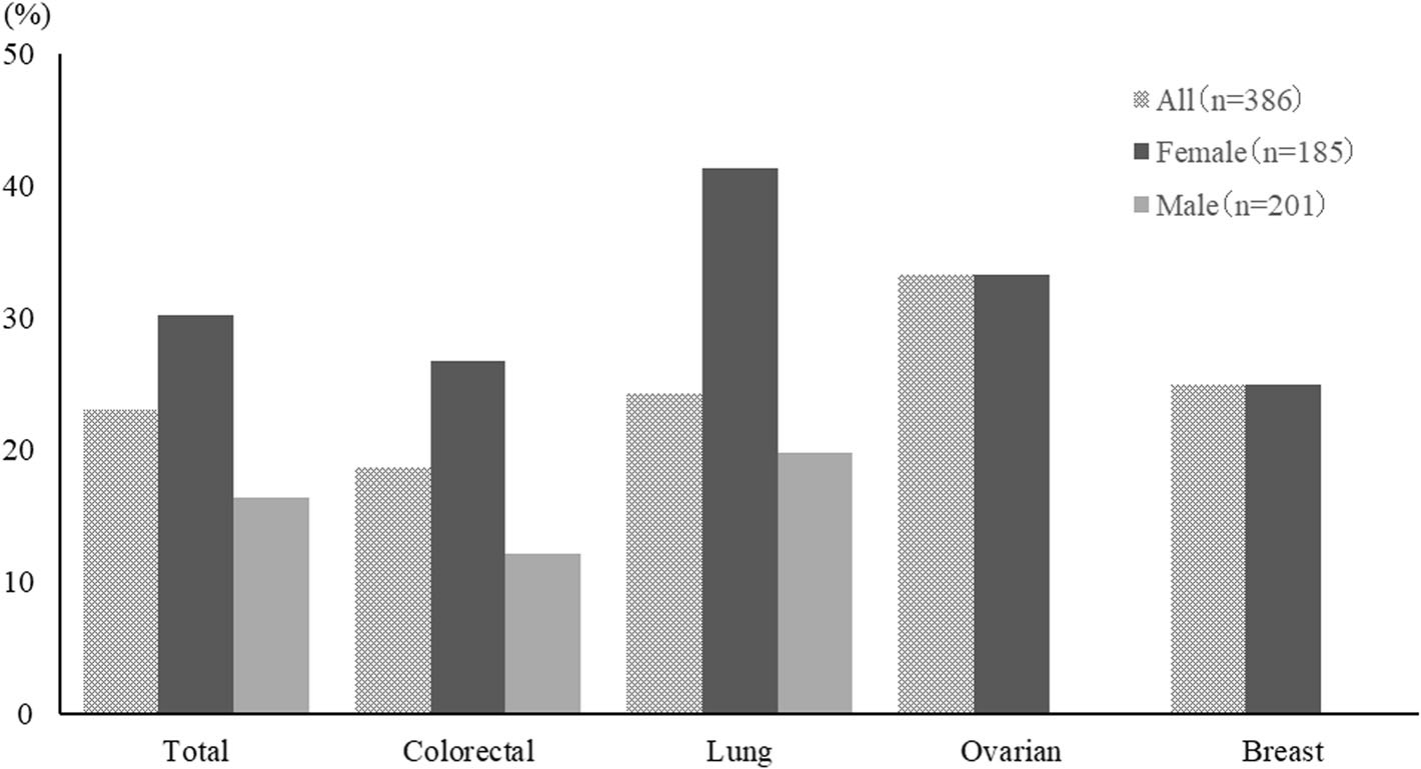
Emetic event rate during the overall phase

Emetic events occurred in 23% of participants overall, with unexpectedly high incidences of emetic episodes in those with colorectal cancer (19%), lung cancer (24%), ovarian cancer (33%), and breast cancer (25%). Emetic episodes occurred significantly more frequently in women (30%) than in men (16%) (Fig. 5).

### Analysis of risk factors

The results of univariate and multivariate logistic regression analyses indicate the degree of CINV risk arising from possible CINV-related factors (Table 3). Male sex, no history of motion sickness, history of drinking habit, and no history of pregnancy-associated vomiting were identified as risk factors for CR and TC in the overall phase, whereas only ages ≥65 years were identified as risk factors for TC in the overall phase.

**Table 3:** Risk factors by univariate and multivariate analysis

	Univariate analysis		multivariate analysis	
	*p* value	OR(95%CI)	*p* value	OR(95%CI)
Complete Response in the overall phase				
Gender:Male vs Female	0.005	1.830[1.204–2.783]		
Age: ≧65 vs < 65	0.090	1.438[0.945–2.187]	0.099	1.448[0.933–2.249]
Motion sickness: Yes vs No	0.003	0.422[0.241–0.740]	0.011	0.469[0.261–0.840]
Drinking habit: Yes vs No	0.032	1.578[1.040–2.394]	0.143	1.394[0.894–2.175]
Smoking History:Yes vs No	0.057	1.523[0.987–2.350]		
Pregnancy associated vomiting: Yes vs Others	0.006	0.516[0.322–0.872]	0.040	0.589[0.355–0.977]
palonosetron vs 1st generation 5HT3RA	0.723	0.927[0.608–1.412]	0.070	0.560[0.300–1.048]
CBDCA-based chemotherapy vs Others	0.469	0.858[0.567–1.299]	0.027	0.494[0.264–0.922]
Total Control in overall phase				
Gender:Male vs Female	0.002	1.911[1.275–2.864]		
Age: ≧65 vs < 65	0.004	1.807[1.205–2.709]	0.006	1.829[1.191–2.808]
Motion sickness: Yes vs No	0.003	0.408[0.226–0.734]	0.031	0.510[0.277–0.939]
Drinking habit: Yes vs No	0.006	1.755[1.172–2.628]	0.059	1.520[0.984–2.348]
Smoking History:Yes vs No	0.194	1.313[0.871–1.979]		
Pregnancy associated vomiting: Yes vs Others	< 0.001	0.375[0.230–0.611]	0.003	0.460[0.275–0.770]
palonosetron vs 1st generation 5HT3RA	0.445	1.172[0.780–1.759]		
CBDCA-based chemotherapy vs Others	0.364	0.831[0.557–1.239]	0.063	0.666[0.434–1.023]

Backward selection method with an entry and exit criteria of 0.2

*OR* Odds ratio, *CI* Confidence interval, *5HT3RA* 5-hydroxytryptamine-3 receptor antagonist, *CBDCA* Carboplatin

Logistic regression analysis revealed history of motion sickness (odds ratio (OR) 0.469 [95% confidence interval (CI): 0.261–0.840], *p* = 0.011), history of pregnancy-associated vomiting (OR 0.589 [95% CI: 0.355–0.977], *p* = 0.040), and CBDCA-based chemotherapy (OR 0.494 [95%CI: 0.264–0.922], *p* = 0.027) as risk factors for CR and history of motion sickness (OR 0.510 [95%CI: 0.373–1.037], *p* = 0.031) and history of pregnancy-associated vomiting (OR 0.460 [95%CI: 0.275–0.770], *p* = 0.003) as risk factors for TC. Additional, Ages ≥65 years is an independent predictive factor for achieving TC.

### Study observation period

Comparisons of the rates of CC over 5-day and 7-day observation periods revealed the following: CBDCA+ETP (82, 77%, respectively), CBDCA+PTX (69, 67%, respectively), and CBDCA+PEM (58, 54%, respectively) for lung cancer; DTX + CPA (75, 70%, respectively) for breast cancer; FOLFOX (63, 63%, respectively) and CAPOX (67, 64%, respectively) for colon cancer; and CBDCA+PTX (53, 51%, respectively) for ovarian cancer. The maximum delta for CC of 5-day and 7-day was 5.9% for CBDCA+ETP, and the average delta was 3.3%.

## Discussion

This study investigated the need for individualization of antiemetic treatment for different chemotherapeutic regimens and risk factors for MEC. In this study, we demonstrated differences in the incidence of CINV between MEC regimens and sexes. The overall CR rate was 64%, with the CR rates for CBDCA+PEM for lung cancer (54%) and CBDCA+PTX for ovarian cancer (51%) being particularly low, followed by FOLFOX (63%), CAPOX (64%), DTX + CPA (70%) and CBDCA+ETP (77%). The overall CR rate was 61.6% for CBDCA-base chemotherapy and 65% for non-CBDCA-base chemotherapy. There was no difference in CR rate between CBDCA-base chemotherapy and non-CBDCA-base chemotherapy. The CR rate in the acute phase was more than 90% for all evaluated regimens. Thus, the poorer overall CR rate reflects the CR rate in the delayed phase. Logistic regression analysis revealed history of motion sickness, history of pregnancy-associated vomiting and CBDCA-based chemotherapy as risk factors for CR and history of motion sickness and history of pregnancy-associated vomiting as risk factors for TC. Additional, Ages ≥65 years is an independent predictive factor for achieving TC.

In our previous study, we reported a CR rate of 68% for palonosetron combined with dexamethasone and aprepitant in patients receiving high emetic risk chemotherapy (HEC) [7]. Therefore, it is necessary to improve the control of CINV in patients receiving CBDCA- or oxaliplatin-based chemotherapy.

We recommend a combination of antiemetic prophylaxis with three antiemetics (5HT3RA, steroids, and NK1RAs) to minimize CINV in patients receiving CBDCA- or oxaliplatin-based chemotherapy because our data indicate that these regimens carry a higher emetic risk than other MECs. Tsuji et al. reported that three antiemetics are more effective than two for the prophylaxis of delayed vomiting in patients with colorectal cancer treated with CAPOX or FOLFOX [8]. However, we found high overall CR rates in men receiving CAPOX (78%), CBDCA+ETP (80%), or CBDCA+PTX for lung cancer (73%). The CR rate in men and women for CBDCA+ETP was 77%, which was much higher than other regimens. Current international guidelines recommend a three-drug combination (NK1RA, a 5HT3RA, and dexamethasone) for patients receiving carboplatin AUC 4 or higher; however, there is insufficient evidence regarding carboplatin AUC 4. Therefore, we believe that further research is necessary to determine the benefits of adding an NK1RA in patients receiving lower doses of carboplatin. Conversely, we found low overall CR rate in men receiving CBDCA+PEM (60.6%). A previous study suggested that the control of CINV in patients treated with CBDCA+PEM receiving two antiemetics was poor. Therefore, it is necessary to consider prophylaxis for CINV for each drug used in combination with CBDCA [9]. In patients receiving oxaliplatin-based chemotherapy, the CR rate for the CAPOX regimen was significantly higher in men (78%) than in women (46%). The CR rate was notably lower in women with colorectal cancer receiving CAPOX than in those with ovarian cancer receiving CBDCA+PTX. We found that female sex strongly contributed to the low CR rate. From the above, two antiemetics may be sufficiently effective for men receiving CBDCA (AUC5) + ETP, CBDCA+PTX for lung cancer, or CAPOX, but the limitation of this study were the high median age and a small number of young people. Additionally, although antiemetic guidelines do not always recommend three antiemetics for oxaliplatin-based regimens, we consider that three antiemetics are necessary for women with colorectal cancer receiving CAPOX.

In a similar observational study of CINV in Japan, Tamura et al. did not report CR, TC, or CC rates or sex comparisons of CR rates [10]. They reported a 16% emetic event rate in the delayed phase in 715 patients receiving MEC, which is notably lower than in our study (23%). This is likely because patients in their study received a triplet antiemetic regimen that included aprepitant, which apparently further reduced the rate of CINV. There is some evidence that adding an NK1RA improves the control of vomiting [11]. Thus, adding an NK1RA to 5HT3RA and dexamethasone may increase therapeutic effectiveness.

The risk factors identified in this study are similar to those reported previously and included well-known risk factors, such as younger age, female sex, history of CINV, and low alcohol consumption [4–6]. However, many similar studies had a stand-alone each carcinoma. The inclusion of patients with colorectal, lung, breast and ovarian cancers in this study may be a strength. We identified non-CBDCA-based chemotherapy as a strong risk factor for CR but not TC that included the assessment of nausea. The onset of nausea in non-CBDCA-based chemotherapy should be carefully monitored. Additionally, age was a strong risk factor for TC but not CR. Young people may be improve CINV by taking specific measures against nausea.

We found no significant difference in the rates of CINV between first-generation 5HT3RA and palonosetron in this study. In a previous study, an open-label, crossover trial was designed to compare the efficacy of palonosetron and ondansetron for MEC [12]. Furthermore, the SENRI trial reported similar results for oxaliplatin-based chemotherapy (FOLFOX or CAPOX) [13].

The observation period in this study was 7 days (168 h), which is longer than the 5 days (120 h) reported in many previous studies of CINV. We found that 5 days of observation resulted in underestimation by up to 5.9% compared with a 7-day observation period. Therefore, we recommend that assessments should continue for around 7 days in future observational studies.

The present study had some limitations. First, its design was neither randomized nor blinded; thus, the present findings should be interpreted within the limitations of an observational study design. Second, there was a bias in the number of patients receiving different chemotherapeutic regimens. Third, NK1RA was not a component of the evaluated antiemetic treatment. Despite these limitations, we identified the incidence of CINV and its associated risk factors in routine clinical practice, rather than in a controlled trial. Additionally, we have presented the characteristics of CINV for different chemotherapeutic regimens.

It has not yet been conclusively demonstrated that a combination of three antiemetics is indicated for all patients receiving MEC. More randomized trials exclusively testing MEC regimens that do not include carboplatin are warranted.

## Conclusion

Our data showed that two antiemetics were insufficient to control CINV in patients receiving CBDCA- or oxaliplatin-based chemotherapy. However, two antiemetics may be sufficiently effective for elderly male patients administered CBDCA (AUC5) + ETP, CBDCA+PTX for lung cancer, or CAPOX. Additionally, we consider that three antiemetics are necessary for women with colorectal cancer receiving CAPOX. Risk factor analysis related to CR showed that CINV prophylaxis for CBDCA-based chemotherapy was generally supportive of the guideline-recommended three antiemetics. However, the control of nausea in patients administered non-CBDCA-based chemotherapy is a key point to note. The further individualization of antiemetic regimens for patients receiving MEC based on both types of chemotherapy regimens and sex is needed. Identified individual risk factors in this study will assist in the development of personalized antiemetic treatments.

## Data Availability

The datasets obtained and/or analyzed during the current study are available from the corresponding author on reasonable request.

## Abbreviations

CINV: Chemotherapy-induced nausea and vomiting
ASCO: American Society of Clinical Oncology
MASCC: Multinational Association of Supportive Care in Cancer
ESMO: European Society of Medical Oncology
NCCN: National Comprehensive Cancer Network
MEC: Moderate emetic risk chemotherapy
5HT3RA: 5-hydroxytryptamine-3 receptor antagonist
NK1RA: Neurokinin 1 receptor antagonist
CBDCA: Carboplatin
ETP: Etoposide
PTX: Paclitaxel
PEM: Pemetrexed
CPA: Cyclophosphamide
DTX: Docetaxel
FOLFOX: Oxaliplatin with fluorouracil and folinic acid
FOLFIRI: Irinotecan with fluorouracil and folinic acid
CAPOX: Capecitabine plus oxaliplatin therapy
CR: Complete response
TC: Total control
CC: Complete control
